# Adaptive Absolute Ego-Motion Estimation Using Wearable Visual-Inertial Sensors for Indoor Positioning

**DOI:** 10.3390/mi9030113

**Published:** 2018-03-06

**Authors:** Ya Tian, Zhe Chen, Shouyin Lu, Jindong Tan

**Affiliations:** 1 School of Information and Electrical Engineering, Shandong Jianzhu University, Jinan 250101, China; chenzhe19930517@163.com (Z.C.); lusy@sdjzu.edu.cn (S.L.); 2 Shandong Provincial Key Laboratory of Intelligent Buildings Technology, Jinan 250101, China; 3 Shandong Provincial Key Laboratory of Intelligent Technology for New Type Man-Machine Interaction and Robot System, Jinan 250101, China; 4 Department of Mechanical, Aerospace, and Biomedical Engineering, The University of Tennessee, Knoxville, TN 37996, USA; tan@utk.edu

**Keywords:** ego-motion estimation, indoor navigation, monocular camera, scale ambiguity, wearable sensors

## Abstract

This paper proposes an adaptive absolute ego-motion estimation method using wearable visual-inertial sensors for indoor positioning. We introduce a wearable visual-inertial device to estimate not only the camera ego-motion, but also the 3D motion of the moving object in dynamic environments. Firstly, a novel method dynamic scene segmentation is proposed using two visual geometry constraints with the help of inertial sensors. Moreover, this paper introduces a concept of “virtual camera” to consider the motion area related to each moving object as if a static object were viewed by a “virtual camera”. We therefore derive the 3D moving object’s motion from the motions for the real and virtual camera because the virtual camera’s motion is actually the combined motion of both the real camera and the moving object. In addition, a multi-rate linear Kalman-filter (MR-LKF) as our previous work was selected to solve both the problem of scale ambiguity in monocular camera tracking and the different sampling frequencies of visual and inertial sensors. The performance of the proposed method is evaluated by simulation studies and practical experiments performed in both static and dynamic environments. The results show the method’s robustness and effectiveness compared with the results from a Pioneer robot as the ground truth.

## 1. Introduction

Recently, with the increasing number of elderly people in many countries, the age-related problems will become increasingly serious, such as hearing loss, sight loss, memory loss and other increased health problems, which definitely lead to a burning issue for all modern societies around the world [1]. Commonly, most aging people with these age-related problems have difficulties in safety and mobility of daily life, especially within unfamiliar environments, so they usually rely on some aiding devices, like a positioning system, to carry out tasks and activities.

As is known, the Global Positioning System (GPS) has been available for a wide variety of navigation applications over the past 50 years because of its high accuracy. Therefore, it is one of the most important parts for positioning and tracking systems and especially plays a key role in outdoor positioning. However, for indoors, and outdoor environments with tall buildings and trees, GPS-based positioning is not suitable due to the unreliable satellite signals. With recent development of miniature sensor technology, more and more researchers have been attracted to developing various wearable electronic aids for aging people to avoid collision and motion risks. However, these aiding devices still have limited functionality and flexibility so that developing a novel wearable indoor positioning system is desirable to make the aging people’s daily life much easier and more convenient.

**Figure 1:**
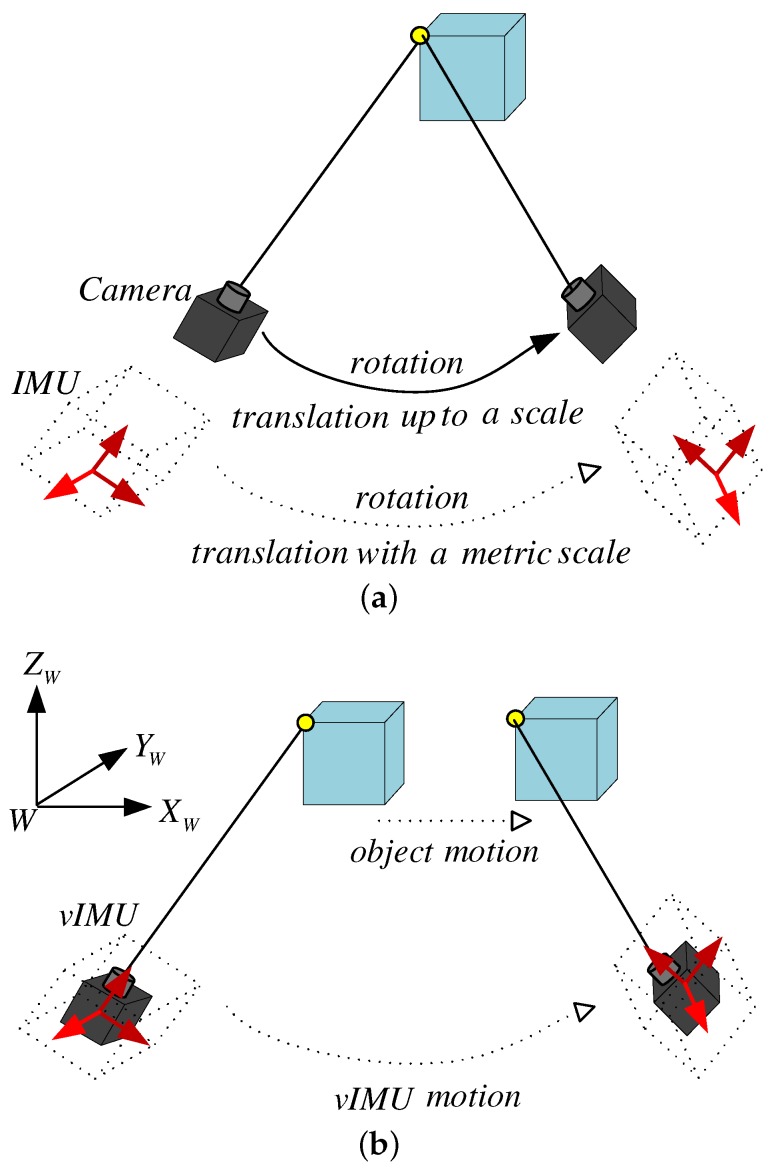
Static and dynamic scene. (**a**) in a static scene, a camera integrated with an IMU can infer the information of the metric scale; (**b**) in a dynamic scene, the problem is how to accurately infer both the vIMU motion and the object motion.

In this paper, we mainly focus on the integration of ego- and ambient-motion tracking in indoor environments using wearable visual-inertial sensors, where global positioning (GPS-denied) is unavailable or inaccurate. The goal of this work is to obtain not only accurate ego-motion estimation, but also the motion of moving object with a metric scale under dynamic scenes. In our work, a moving visual IMU (Inertial Measurement Unit) system (vIMU) is developed to observe both a 3D static scene as shown in Figure 1a and a 3D dynamic scene as shown in Figure 1b. Rotational and translational motion is estimated individually by visual and inertial sensors. Outliers from visual estimations due to the variety of dynamic indoor environment are rejected via our proposed adaptive orientation method using MARG (Magnetic, Angular Rate and Gravity) sensors [2]. Moreover, a concept of “virtual camera” is presented to consider the motion area of each moving object as if a static object were observed by a “virtual camera”, while the motion of the “real camera” is estimated by the extracted features from the static background. In particular, considering of the different sampling rates of visual and inertial sensors and the scale ambiguity in monocular camera tracking, we propose a multi-rate linear Kalman-filter (MR-LKF) to integrate visual and inertial estimations.

**Figure 2:**
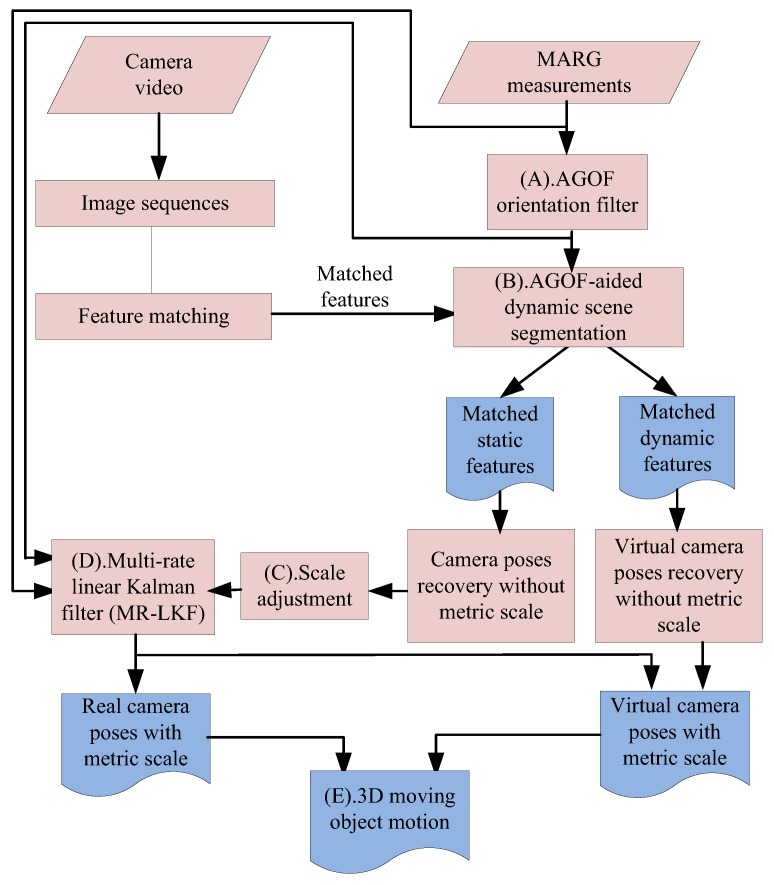
The main framework of the proposed method.

The main contributions of this paper are summarized as follows: (1) a novel method for dynamic scene segmentation based on AGOF-aided (Adaptive-Gain Orientation Filter) homography recovery constraint and epipolar geometry constraint shown as process (B) in Figure 2; (2) a new concept of “virtual camera” for robust ego- and ambient-estimation in dynamic environments; and (3) an MR-LKF fusion method for solving the problems of two different sampling rates and scale ambiguity.

## 2. Related Work

In recent years, with the development of technology in computer vision, more and more researchers have been attracted to develop monocular visual-based localization algorithms based on the theory of structure from motion (SFM) [3,4,5,6]. However, there are two main problems with monocular visual-based localization algorithms. One is the triangulation problem, which can only be enabled in at least two views where the 3D scene is commonly assumed to be static. If there are other objects moving in the 3D scene, which is referred to as the dynamic 3D scene, the rule of triangulation will fail unless some constraints are further applied [7]. The other is the visual scale problem, which is usually lost when projecting a 3D scene on a 2D imaging plane. The most common approach for doing so is stereo vision [8,9]. Although these systems work well in many environments, stereo vision is fundamentally limited by two specific cameras. In addition, the structure of 3D environment and the motion of camera could be recovered from a monocular camera using structure from motion (SFM) techniques [10,11,12,13,14], but they are up to an arbitrary scale. Methods appearing in structure from motion to infer the scale of the 3D structure is to place an artificial reference with a known scale into the scene. However, it limits its applications to place a marker before the 3D reconstruction .

In the past 10 years, the integration of visual and inertial sensors has shown more significant performance than a single sensor system, especially in positioning and tracking systems [8,15,16,17] due to their complementary properties [18]. Inertial sensors provide good signals with high-rate motions in the short term but suffer from accumulated drift due to the double integration during the estimation of position. On the contrary, visual sensors offer accurate ego-motion estimation with low-rate motion in the long term, but are sensitive to blurred features during unpredicted and fast motions [19]. Therefore, recently, these complementary properties have been utilized by more and more researchers as the basic principle for integrating visual and inertial sensors together. Moreover, the inertial sensors can not only be small in size, light weight and low in cost, but also easily adopt wireless communication technologies, so it is much easier for people to wear them. This is why we call them “wearable” inertial sensors.

In general, the Kalman filter (KF) is a common and popular algorithm for sensor fusion and data fusion, which is an efficient recursive filter and widely used in many applications. In recent years, more and more researchers have been attracted to develop novel Kalman-filter-based algorithms to deal with structural systems. In structural systems, the states including displacements and velocities are difficult or sometimes impossible to measure, so a variety of novel Kalman filters have been developed from Kalman’s original formulation by accounting for non-stationary unknown external inputs and theoretical investigation of observability, stability and associated advancements [20,21,22,23]. To our knowledge, nonlinear Kalman filter techniques are usually applied to almost all of the inertial-visual fusion algorithms, such as extended KF, unscented KF, etc. [8,17,24,25,26], because a large state vector and a complex nonlinear model are required when both the orientation and the position are optimized in the same process. However, an unacceptable computational burden would be imposed because of so many recursive formulas. Moreover, the linear approximations of EKF may result in non optimal estimates. Although [27] proposed a modified linear Kalman filter to perform the fusion of inertial and visual data, the accurate orientation estimates were based on the assumption of gyroscope measurements trusted for up to several minutes. In [28], the authors proposed a novel fusion algorithm by separating the orientation fusion and the position fusion process, while the orientation estimation could only be robust for a static or slow movement without magnetic distortions using the method proposed in [29]. In contrast, in this paper, the orientation is firstly estimated by our previously proposed orientation filter in [2] only from inertial measurements. Our orientation filter can not only obtain the robust orientation in real time for both extra acceleration and magnetic distortions, but also eliminate the bias and noise in angular velocity and acceleration. In addition, the sampling rates for visual and inertial sensors are inherently different. As a result, an efficient inertial-visual fusion algorithm, called multi-rate AGOF/Linear Kalman filter (MR-LKF), is proposed to separate the orientation and the position estimation; thus, this results in a small state vector and a linear model. A summary of the related work on inertial-visual integration is presented in Table 1.

## 3. Sensors

This section introduces some preliminary notations and definitions for the camera and integrated visual-inertial (vIMU) system. For brevity and clarity, Table 2 gives the definitions of mathematical symbols and variables.

### 3.1. Camera

#### 3.1.1. Camera Model

**Figure 3:**
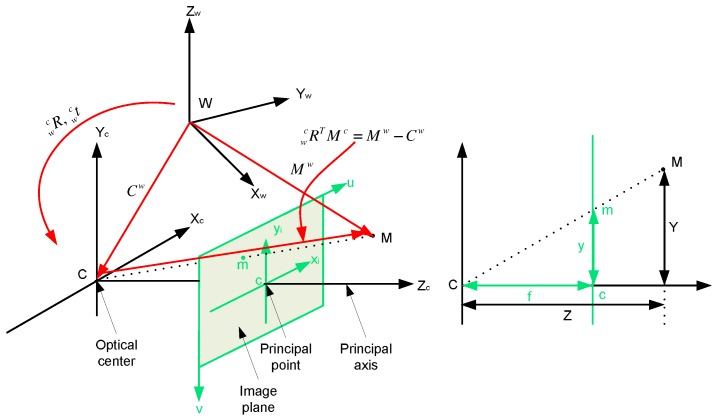
**left**: the relationship between the camera and image coordinates and between the camera and world coordinates; **right**: side view of the left figure is used to derive the relationship between the camera and image coordinates based on the principle of similarity.

A camera is a mapping between the 3D world and a 2D image, so the most specialized and simplest camera modle, called the basic pinhole camera model [10], is used here to deduce the mapping between a point in 2D image coordinate system and a point in 3D camera coordinate system. Under this model, a 3D point $M^{c}={\left(X,Y,Z\right)}^{T}$ in the camera coordinate system *c* is mapped to the 2D point $m^{i}={\left(x,y\right)}^{T}$ in the image coordinate system *i*, which is located on the image plane (Z=f). A line joining the point $M^{c}$ to the center of projection (called camera centre) meets the image plane illustrated in Figure 3. Based on triangle similarity, the relationship between $m^{i}$ and $M^{c}=\left(X,Y,Z\right)$ is given in Label (1):

(1) $$\begin{array}{ccc} x & = & fX/Z,\\ y & = & fY/Z, \end{array}$$

where *f* denotes the focal length. Based on the representation of homogeneous coordinates, Label (1) can be rewritten as a linear mapping using a matrix representation denoted in Label (2):

(2) $$\begin{array}{c} Z*\left[\begin{array}{c} x\\ y\\ 1 \end{array}\right]=\left[\begin{array}{cccc} f & 0 & 0 & 0\\ 0 & f & 0 & 0\\ 0 & 0 & 1 & 0 \end{array}\right]*\left[\begin{array}{c} X\\ Y\\ Z\\ 1 \end{array}\right]. \end{array}$$

By introducing a non-zero homogenous scaling factor *s*, Label (2) can be rewritten in Label (3):

(3) $$\begin{array}{c} \left[\begin{array}{c} x\\ y\\ 1 \end{array}\right]=s*\left[\begin{array}{cccc} f & 0 & 0 & 0\\ 0 & f & 0 & 0\\ 0 & 0 & 1 & 0 \end{array}\right]*\left[\begin{array}{c} X\\ Y\\ Z\\ 1 \end{array}\right]. \end{array}$$

#### 3.1.2. Intrinsic Camera Parameters

Usually, most of the current imaging systems use pixels to measure image coordinates where the origin of the pixel coordinate system is located at the top-left pixel of the image. Therefore, in order to describe a projected point in the pixel coordinate system, the intrinsic camera parameters have to be taken into account. If $m^{p}={\left(u,v\right)}^{T}$ represents the 2D point in the pixel coordinate system *p* corresponding to the 2D point $m^{i}={\left(x,y\right)}^{T}$ in image coordination system *i*, the relationship between $m^{p}={\left(u,v\right)}^{T}$ and $m^{i}={\left(x,y\right)}^{T}$ can be rewritten in Label (4):

(4) $$\begin{array}{ccc} u & = & k_{x}x+c_{x},\\ v & = & k_{y}y+c_{y}, \end{array}$$

where $k_{x}$ and $k_{y}$, respectively, represent the number of pixels per unit of length in the direction of *x* and *y*. Based on Label (4) and the representation of homogeneous coordinates, the correlation of $(m^{i}={\left(x,y\right)}^{T}$ and $m^{p}={\left(u,v\right)}^{T}$ can be easily denoted in Label (5) using a matrix representation:

(5) $$\begin{array}{c} \left[\begin{array}{c} u\\ v\\ 1 \end{array}\right]=\left[\begin{array}{ccc} k_{x} & 0 & c_{x}\\ 0 & k_{y} & c_{y}\\ 0 & 0 & 1 \end{array}\right]*\left[\begin{array}{c} x\\ y\\ 1 \end{array}\right]. \end{array}$$

Depending on Labels (3) and (5), we can easily express the mapping between a 3D point $M^{c}=\left(X,Y,Z\right)$ in the camera frame and its corresponding 2D point $m^{p}={\left(u,v\right)}^{T}$ in the pixel frame using the camera intrinsic calibration matrix *K* as shown in the following equation:

(6) $$\begin{array}{c} \left[\begin{array}{c} u\\ v\\ 1 \end{array}\right]=\left[\begin{array}{ccc} k_{x} & 0 & c_{x}\\ 0 & k_{y} & c_{y}\\ 0 & 0 & 1 \end{array}\right]*\left[\begin{array}{c} x\\ y\\ 1 \end{array}\right]\\ =s*\left[\begin{array}{cccc} f_{x} & 0 & c_{x} & 0\\ 0 & f_{y} & c_{y} & 0\\ 0 & 0 & 1 & 0 \end{array}\right]*\left[\begin{array}{c} X\\ Y\\ Z\\ 1 \end{array}\right]=s*K*\left[\begin{array}{c} X\\ Y\\ Z\\ 1 \end{array}\right], \end{array}$$

where $f_{x}$ and $f_{y}$, called focal distances, can be respectively obtained by using $k_{x}$ and $k_{y}$ multiplied by the focal length *f*.

#### 3.1.3. Extrinsic Camera Parameters

Generally, 3D points are not expressed in the moving camera coordinate system *c* but in a fixed reference frame, called the world coordinate system *w*. The relationship between those coordinate systems can be given by a rigid transformation consisting of a rotation ${}_{w}^{c}R$ and a translation ${}_{w}^{c}t$ called the extrinsic camera parameters or the camera pose. This is illustrated on the right side of Figure 3. The mapping of a point $M^{w}$ expressed in the world frame to a point $M^{c}$ expressed in the camera frame can be denoted as follows:

(7) $$\begin{array}{ccc} M^{c} & = & {}_{w}^{c}R\left(M^{w}-C^{w}\right), \end{array}$$

where $C^{w}$ is the position of the camera center in the world frame. Label (7) can be rewritten in another commonly used form as illustrated in Lable (8):

(8) $$\begin{array}{ccc} M^{c} & = & {}_{w}^{c}RM^{w}+{}_{w}^{c}t, \end{array}$$

where the rotation ${}_{w}^{c}R$ is pre-estimated only from inertial sensors and then used for calculating the translation ${}_{w}^{c}t$ denoted as ${}_{w}^{c}t=-{}_{w}^{c}RC^{w}$. By introducing homogeneous coordinates, Label (8) can be expressed as a linear operation shown in Label (9):

(9) $$\begin{array}{c} \left[\begin{array}{c} X^{c}\\ Y^{c}\\ Z^{c}\\ 1 \end{array}\right]=\left[\begin{array}{cc} {}_{w}^{c}R & {}_{w}^{c}t\\ 0_{3}^{T} & 1 \end{array}\right]*\left[\begin{array}{c} X^{w}\\ Y^{w}\\ Z^{w}\\ 1 \end{array}\right], \end{array}$$

where ${}_{w}^{c}R$ and ${}_{w}^{c}t$ are the camera’s extrinsic parameters.

#### 3.1.4. From World to Pixel Coordinates

By combining the forward transformations given in Label (6) and Label (9), the expected pixel location $m^{p}$ can be computed from each point $M^{w}$ using Label (10):

(10) $$\begin{array}{c} \left[\begin{array}{c} u\\ v\\ 1 \end{array}\right]=s*K*\left[\begin{array}{c} X^{c}\\ Y^{c}\\ Z^{c}\\ 1 \end{array}\right]=s*K*\left[\begin{array}{cc} {}_{w}^{c}R & {}_{w}^{c}t\\ 0_{3}^{T} & 1 \end{array}\right]*\left[\begin{array}{c} X^{w}\\ Y^{w}\\ Z^{w}\\ 1 \end{array}\right], \end{array}$$

so the mapping can be simply expressed as $m^{p}∼PM^{w}$, where the matrix $P=K[{}_{w}^{c}R|{}_{w}^{c}t]$ is called the camera projection matrix, and ∼ means equivalence up to a scale factor.

### 3.2. Visual IMU System

**Figure 4:**
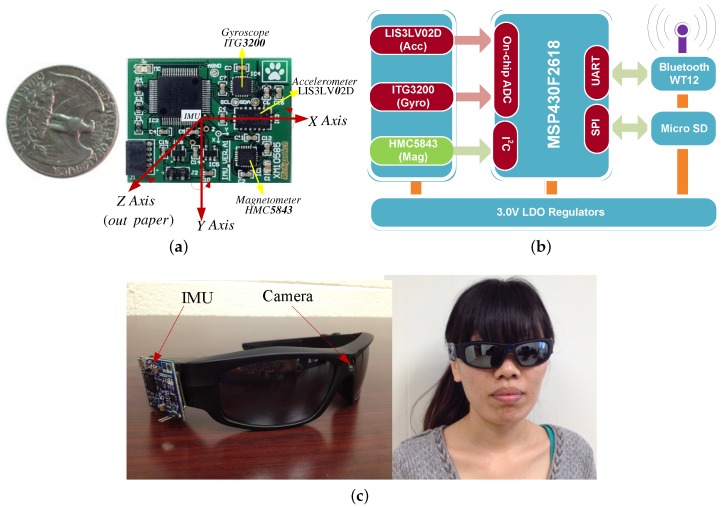
Prototype of MARG sensor and wearable vIMU system. (**a**) the developed MARG sensor; (**b**) hardware configuration of MARG sensor; (**c**) the wearable vIMU system.

A wearable visual IMU (vIMU) system is shown in Figure 4c. It integrates a camera and a newly developed MARG sensor together on a sunglass, so it is convenient for people to wear. The camera has a feature of 170 degree wide-angle, HD (High Definition) camera lens with 5.0 Mage 720P. Figure 4a shows the prototype of MARG sensor, which contains a tri-axis accelerometer (LIS3LV02D), a tri-axis gyroscope (ITG3200) and a tri-axis magnetometer (HMC5843) in a small sensor package. All signals are transmitted through Bluetooth. Moreover, an processor (TI MSP430F2618) is embedded in the small chip for convenient computation. The hardware configurations of the MARG sensor is shown in Figure 4b.

In order to integrate the measurements from different sensors, their measurements have to be timely and spatially synchronized due to each physical sensor providing measurements in its own time and spatial reference. The proposed vIMU system provides timely synchronized image sequences and inertial readings. The sample rate of MARG sensor is 60 Hz and the sample rate of camera can be lower due to the accurate baseline from epipolar geometry constraint. The related coordinate systems connected to the camera and the MARG sensor have already been presented in our previous work [2,30].

## 4. Motion Estimation

In this section, an adaptive method is presented to estimate motion from visual measurements with the help of inertial measurements. Inertial motion estimation outputs the real-time orientation using an adaptive-gain orientation filter (AGOF) from our previous work [2], which aids visual motion estimation to not only segment dynamic scenes, but also compute the camera transformation from corresponding features between consecutive images.

### 4.1. AGOF-Aided Dynamic Scene Segmentation

The SIFT (Scale-Invariant Feature Transform) algorithm is selected to generate a SIFT descriptor corresponding to each key-point [31] and then all 2D matched feature points are obtained. The goal of our work is to propose a robout algorithm to classify these matched feature points. As a result, different groups of matched featuer points are used to recover the corresponding motions. In this subsection, we present the sorted method for matched feature points: AGOF-aided homography recovery constraint and epipolar geometry constraint.

#### 4.1.1. Homography Recovery

When the camera undergoes a pure translation, a general motion of camera can be transformed to a special motion with the help of the preestimated robust orientation from our AGOF filter. Usually, there are two special cases: one is parallel to the image plane and the other is perpendicular to the image plane.

**Figure 5:**
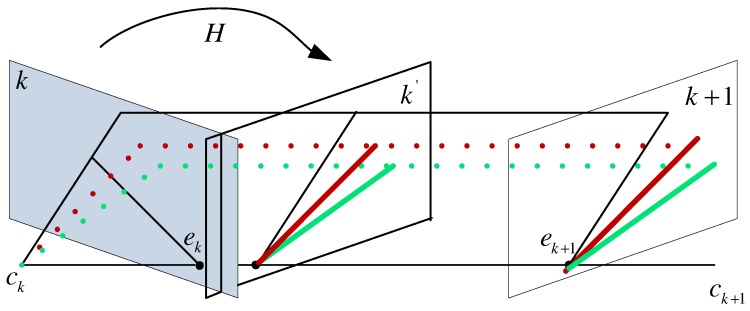
Homography recovery under a general motion of camera.

As shown in Figure 5, the homography *H* can recover rotation between two consecutive images because it directly connects the corresponding 2D points of a 3D point. If the camera intrinsic parameter *K* and the rotation *R* are known, the homography *H* can be directly obtained using Label (11):

(11) $$\begin{array}{ccc} m_{k+1}^{T} & = & Hm_{k},\\ H & = & KRK^{-1}, \end{array}$$

where $m_{k}=\left(u_{k},v_{k}\right)$ and $m_{k+1}=\left(u_{k+1},v_{k+1}\right)$ are corresponding 2D points in two consecutive frames *k* and k+1 of a 3D point M. As we mentioned previously, the rotation *R* can be preestimated, so a bunch of motion lines, which connect the corresponding 2D matched feature points of a 3D point, are obtained. These lines can be sorted by computing the slope of them or checking if they can intersect at the same point called “epipole”. The slope of motion line ρ can be expressed in Label (12) according to $m_{k}$ and $m_{k+1}$:

(12) $$\begin{array}{cc} \rho & =\frac{u_{k+1}-u_{k}}{v_{k+1}-v_{k}},\;\;\mathrm{if}\;∥v_{k+1}-v_{k}∥\neq 0. \end{array}$$

If $∥v_{k+1}-v_{k}∥$ equals 0, then the real camera moves along *x*-axis of the camera coordinate system.

#### 4.1.2. Epipolar Geometry

**Figure 6:**
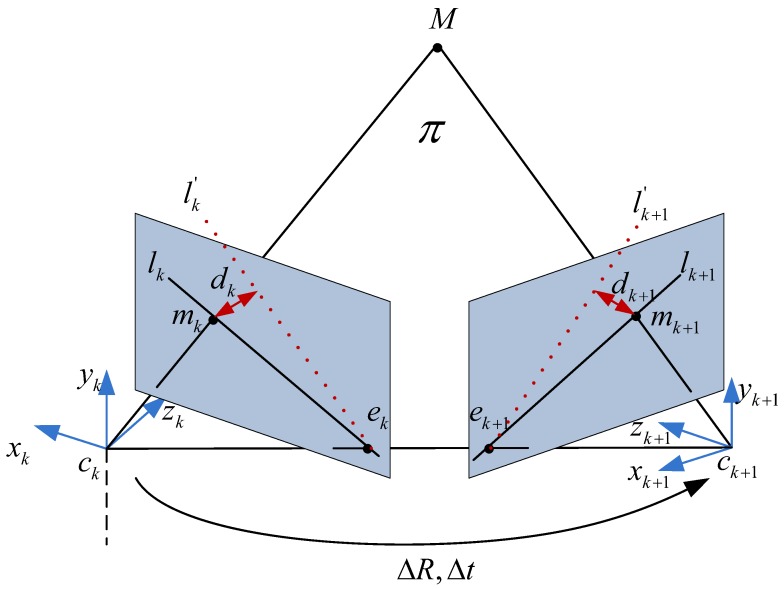
Epipolar geometry.

According to the definition in reference [10], two epipolar lines can be obtained in Label (13) based on the optical flows:

(13) $$\begin{array}{ccccc} l_{k} & = & e_{k}\times m_{k} & = & {\left[e_{k}\right]}_{\times }m_{k},\\ l_{k+1} & = & e_{k+1}\times m_{k+1} & = & {\left[e_{k+1}\right]}_{\times }m_{k+1}, \end{array}$$

where $e_{k}$ and $e_{k+1}$ are the epipoles; ${\left[e_{k}\right]}_{\times }$ and ${\left[e_{k+1}\right]}_{\times }$ are 3×3 skew-symmetric matrixes; $l_{k}$ and $l_{k+1}$ respectively denote lines connecting e and m in frame *k* and frame k+1 respectively as shown in Figure 6. Moreover, based on the constraint of epipolar geometry as depicted in Label (14), two epipolar lines $l_{k}^{{}^{\prime }}$ and $l_{k+1}^{{}^{\prime }}$ could be inferred from the fundamental matrix F as shown in Label (15):

(14) $$\begin{array}{c} m_{k+1}^{T}Fm_{k}=0, \end{array}$$

(15) $$\begin{array}{ccccccc} l_{k}^{{}^{\prime }} & = & F^{T}m_{k+1} & , & l_{k+1}^{{}^{\prime }} & = & Fm_{k}. \end{array}$$

Based on two constraints of optical flow and epipolar geometry for static points, we can obtain $l_{k}≅l_{k}^{{}^{\prime }}$ and $l_{k+1}≅l_{k+1}^{{}^{\prime }}$, where ≅ means up to a scale factor. Nevertheless, the constrain of epipolar geometry will be not satisfied if the points belong to moving objectgs. Therefore, for feature points from moving objects in the scene, the distance from the 2D point to the corresponding epipolar line is chosen to evaluate how discrepant this epipolar line is, and it can be derived in Label (16) from the constraint of epipolar geometry:

(16) $$\begin{array}{ccc} d_{k} & = & \frac{m_{k}^{T}\left(F^{T}m_{k+1}\right)}{{∥F^{T}m_{k+1}∥}^{2}},\\ d_{k+1} & = & \frac{m_{k+1}^{T}\left(Fm_{k}\right)}{{∥Fm_{k}∥}^{2}}. \end{array}$$

In general, the distance for a static point is non-zero due to image noises and estimation errors of epipolar geometry. Actually, the larger the distance, the more confidently the 3D point is considered to be part of a moving object.

### 4.2. Real and Virtual Camera Motion Recovery

**Figure 7:**
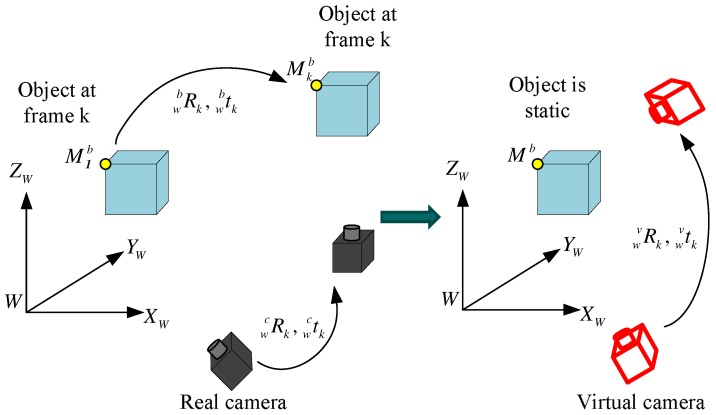
A concept of virtual camera.

Based on the matched 2D feature points from a moving object viewed by a moving camera, the recovered poses actually reflect the combined motion of the moving object and the moving camera. For better understanding, a novel concept of a “virtual” camera is proposed to consider as if the object were static observed by a “virtual” moving camera in comparison with the “real” camera as depicted in Figure 7. This section will emphasize how to recover the motion of real and virtual camera.

#### 4.2.1. Relative Motion Recovery for Real Camera

After dividing 2D matched feature points based on two pre-presented constraints, the essential matrix E can be derived from the fundamental matrix F and the camera’s intrinsic parameter matrix *K* using Labels (14) and (17):

(17) $$\begin{array}{c} E=K^{T}FK. \end{array}$$

As we know, the relative translation Δt and rotation ΔR of camera can be obtained from the essential matrix E, so Δt and ΔR could differentiate the relative motion of camera from the absolute motion of camera. Authors in [10] retrieved the camera matrices from the essential matrix E using $E={\left[t\right]}_{\times }R$, so the relative rotation ΔR and the relative translation Δt, as shown in Figure 6, can be recovered from E by using Labels (18) and (19) based on the method proposed in [32]:

(18) $$\begin{array}{ccc} \Delta t\;\Delta t^{T} & = & \frac{1}{2}Trace\left(EE^{T}\right)I-EE^{T},\\ (\Delta t\cdot \Delta t)\Delta R & = & E^{*T}-\Delta t\times E, \end{array}$$

where $E^{*}$ is E’s cofactor and I is a 3×3 identity matrix. As a result, two solutions $\Delta t_{1}$ and $\Delta t_{2}$ could be obtained for Δt by finding the largest row of matrix $T=\Delta t\;\Delta t^{T}$ as shown in Label (19):

(19) $$\begin{array}{ccc} \Delta t & = & \pm T{\left(i,:\right)}^{T}/\sqrt{T(i,i)}, \end{array}$$

where T(i,i) is the largest element of diagonal of matrix T (i=1,2,3). Therefore, the camera matrix has only two different solutions: $P_{1}=\left[\Delta R|\Delta t_{1}\right]$ and $P_{2}=\left[\Delta R|\Delta t_{2}\right]$ due to pre-estimated accurate ΔR in [2]. Here, we use the rule that a reconstructed 3D point should be in front of the camera between two consecutive views to check which one of these two solutions is satisfied.

Finally, a refining process called “Bundle Adjustment” is used to optimize the parameters of the relative motion Δt and the optical characteristics of the camera *K*, according to a nonlinear least square solution to minimize the total reprojection errors of all points in two consecutive images at *k* and k+1 as shown in Label (20):

(20) $$\begin{array}{ccc} \epsilon & = & {\left[\epsilon_{k},\epsilon_{k+1}\right]}^{T},\\ \epsilon_{k} & = & \sum_{i}∥{}^{i}m_{k}-K\left[eye\left(3\right)|0_{3\times 1}\right]{}^{i}M∥,\\ \epsilon_{k+1} & = & \sum_{i}∥{}^{i}m_{k+1}-K\left[\Delta R|\Delta t\right]{}^{i}M∥, \end{array}$$

where ${}^{i}m_{k}$ represents the *i*-th 2D point in the image coordinate at frame *k* and ${}^{i}M$ is the corresponding 3D point.

#### 4.2.2. Relative Motion Recovery for Virtual Camera

According to the pre-proposed concept of virtual camera, the motion of virtual camera is actually the combination motion of real camera and moving object. In addition, the intrinsic parameters of virtual camera is the same as those of real camera, but the motion of virtual camera is different from that of real camera with the presence of moving objects.

Meanwhile, the relative motion of virtual camera can be obtained by using the similar method as the real camera in Section 4.2.1. The only difference is that the relative rotation does not need to be recovered for the real camera because the real camera is rigidly attached with the IMU and the rotation of real camera can be pre-estimated from IMU-only.

#### 4.2.3. Scale Adjustment

**Figure 8:**
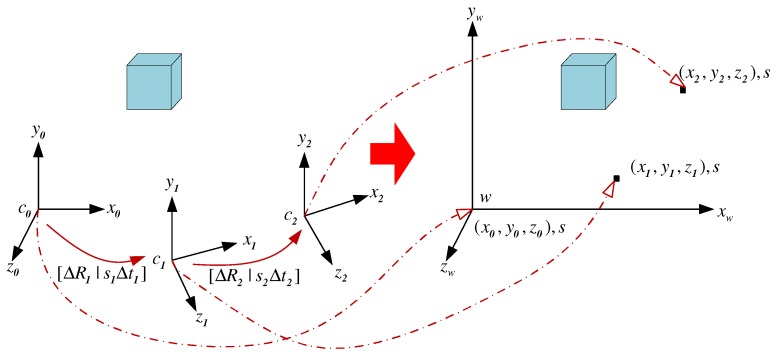
Unified scale recovery from videos.

The baseline Δt, recovered from E based on Label (19), can only have available direction because the camera motion is only estimated up to a scale. This is a so-called scale problem in monocular vision. Since there are multiple frames, the baseline estimated between each pair of consecutive frames is only determined up to an unknown and different scale factor as shown in Figure 8.

In order to obtain a scale consistent trajectory estimation of the camera motion, the scale for each camera motion between two consecutive frames needs to be adjusted so that only one global scale parameter remains unknown. This global scale defines the true size of the reconstructed 3D structure and can be recovered if the information about the real world is introduced. In this subsection, an inertial measurement unit, which consists of three-axis accelerometer, gyroscope and magnetometer, is used to infer the information about the real world. Section 5 will introduce the estimation of this absolute global scale in details.

For adjusting the scale, the method proposed in [33] will be employed in this subsection, where the scale is computed in closed form with a 1-point algorithm. Given the scale free motion estimation ([ΔR|Δt]) of the camera from frame *k* to frame k−1, the feature matches between frame k−1 and frame *k* ($m={\left(x,y,1\right)}^{T}=K^{-1}*{\left(u,v,1\right)}^{T}$), and the reconstructed 3D points ($M={\left(X,Y,Z,1\right)}^{T}$) obtained from three consecutive frames k−2, k−1 and *k* , the scale can be adjusted as follows:

(21) $$\begin{array}{ccc} m & = & [\Delta R|s_{k}\Delta t]M, \end{array}$$

where $s_{k}$ is the scale ratio that relates the baseline between camera frame k−2 and frame k−1 and the baseline between camera frame k−1 and frame *k*. Label (21) can be rewritten as $As_{k}=b$, where A and b are vectors. The vector A contains one constraint per row $\Delta t_{x}-\Delta t_{z}x$, defined by one 2D ∼ 3D correspondence. The vector b is defined as: $(\Delta r_{1}-\Delta r_{3}x)X$ where $\Delta r_{1}$ is the first row of ΔR. Then, the scale $s_{k}$ is solved by using SVD (Singular Value Decomposition) [10] for obtaining a solution in the least square sense as:

(22) $$\begin{array}{ccc} s_{k} & = & \frac{A^{T}b}{A^{T}A}. \end{array}$$

Though only one 2D∼3D correspondence is needed to solve the scale parameter, all available correspondences are used in this paper for robustness.

#### 4.2.4. Camera Absolute Motion Recovery

Usually, the camera absolute poses, which are relative to the world coordinate, are essential to be obtained for motion estimation. However, from the 2D matched points, we can derive the relative rotation ΔR and translation Δt between two consecutive frames. If ($R_{k},t_{k}$) and ($R_{k+1},t_{k+1}$) respectively represent the absolute rotation and translation of camera for frame *k* (k=1,2,⋯) and k+1, then a static 3D point M can be easily expressed between the camera frame and the world frame as shown in Labels (23) and (24):

(23) $$\begin{array}{ccc} M_{k}^{c} & = & R_{k}M_{k}^{w}+t_{k}, \end{array}$$

(24) $$\begin{array}{ccc} M_{k+1}^{c} & = & R_{k+1}M_{k+1}^{w}+t_{k+1}. \end{array}$$

The position of M will not be changed from frame *k* to frame k+1 because M is a static point and meanwhile the world frame does not move. In other words, we can easily define $M_{k}^{w}=M_{k+1}^{w}=M^{w}$, then $M^{w}$ can be derived from Label (23) as $M^{w}=R_{k}^{T}\left(M_{k}^{c}-M_{k}\right)$. Thus, Label (25) is obtained by substituting $M^{w}$ for $M_{k+1}^{w}$ in Label (24):

(25) $$\begin{array}{ccc} M_{k+1}^{c} & = & R_{k+1}R_{k}^{T}M_{k}^{c}-R_{k+1}R_{k}^{T}t_{k}+t_{k+1}\\ & = & \Delta RM_{k}^{c}+\Delta t, \end{array}$$

with $\Delta R=R_{k+1}R_{k}^{T}$ and $\Delta t=t_{k+1}-R_{k+1}R_{k}^{T}t_{k}$. Inversely, given (ΔR,Δt) and ($R_{k},t_{k}$), the camera’s absolute poses at frame k+1 cane be easily solved by using Label (26):

(26) $$\begin{array}{ccc} R_{k+1} & = & \Delta RR_{k},\\ t_{k+1} & = & \Delta t+\Delta Rt_{k}. \end{array}$$

### 4.3. Motion Estimation for 3D Object

In this section, the motion of 3D objects in the world frame will be estimated from the motion of real camera and virtual camera. Assuming that a 3D point $M_{k}^{b}$ is attached to a moving object as depicted in the left of Figure 7, $M_{k}^{b}$ can be derived from the initial position $M_{1}^{b}$ according to the motion of rigid object( ${}_{w}^{b}R_{k}$ and ${}_{w}^{b}t_{k}$) Label (27):

(27) $$\begin{array}{ccc} M_{k}^{b} & = & {}_{w}^{b}R_{k}M_{1}^{b}+{}_{w}^{b}t_{k}, \end{array}$$

where superscript *b* indicates the point M is attached to the moving object and subscript *k* denotes the point is viewed at frame *k*. It is clearly seen that the static rigid object is a special case where ${}_{w}^{b}R_{k}=I$ and ${}_{w}^{b}t_{k}=0$.

Based on the motion of real camera ${}_{w}^{c}R_{k}$ and ${}_{w}^{c}t_{k}$ at frame *k*, we can use Label (28) to obtain the 3D position of a point from the world frame to the current real camera frame:

(28) $$\begin{array}{ccc} M_{k}^{c} & = & {}_{w}^{c}R_{k}M_{k}^{b}+{}_{w}^{c}t_{k}. \end{array}$$

Combining Labels (27) and (28), the 3D position of point with respect to the *k*-th camera can be easily derived in Label (29):

(29) $$\begin{array}{ccc} M_{k}^{c} & = & \left({}_{w}^{c}R_{k}{}_{w}^{b}R_{k}\right)M_{1}^{b}+\left({}_{w}^{c}R_{k}{}_{w}^{b}t_{k}+{}_{w}^{c}t_{k}\right). \end{array}$$

As aforementioned, the special case with ${}_{w}^{b}R_{k}=I$ and ${}_{w}^{b}t_{k}=0$ can be thought as the static object observed by a moving camera, which can simplify Label (29) to be Label (8). Actually, the definition of the “virtual” camera originates from Label (29), which denotes a static object ($M_{1}^{b}=M_{k}^{b}$) viewed by a moving camera as shown in the right of Figure 7. Therefore, the motion of “virtual” camera (${}_{w}^{v}R_{k}$, ${}_{w}^{v}t_{k}$) at frame *k* can be denoted in Label (30):

(30) $$\begin{array}{ccc} {}_{w}^{v}R_{k} & = & {}_{w}^{c}R_{k}{}_{w}^{b}R_{k},\\ {}_{w}^{v}t_{k} & = & {}_{w}^{c}R_{k}{}_{w}^{b}t_{k}+{}_{w}^{c}t_{k}. \end{array}$$

It is clearly seen that the initial point has ${}_{w}^{b}R_{k}=I$ and ${}_{w}^{b}t_{k}=0$ in frame 1, so the motion of virtual camera has the same motion as the real camera at frame 1: ${}_{w}^{v}R_{1}={}_{w}^{c}R_{1}=I$ and ${}_{w}^{v}t_{1}={}_{w}^{c}t_{1}=0$. During the following frames, the virtual camera’s motion differs from the real camera’s motion because of the motion of rigid objects.

As a result, the object pose (${}_{w}^{b}R_{k}$, ${}_{w}^{b}t_{k}$) can be derived by using Label (30) based on the real camera’s motion (${}_{w}^{c}R_{k}$, ${}_{w}^{c}t_{k}$) and the virtual camera’s motion (${}_{w}^{v}R_{k}$, ${}_{w}^{v}t_{k}$):

(31) $$\begin{array}{ccc} {}_{w}^{b}R_{k} & = & {\left({}_{w}^{c}R_{k}\right)}^{-1}{}_{w}^{v}R_{k},\\ {}_{w}^{b}t_{k} & = & {\left({}_{w}^{c}R_{k}\right)}^{-1}\left({}_{w}^{v}t_{k}-{}_{w}^{c}t_{k}\right). \end{array}$$

## 5. Multi-Rate Linear Kalman Filter

As we mentioned previously, the main problem of monocular vision is scale ambiguity. The inertial sensors can infer the position with absolute metric unit from the accelerometer, which suffers from the accumulated drift for long-term tracking. Therefore, the combination of monocular visual and inertial data is proposed in this paper to solve the scale ambiguity. In the state-of-the-art literature [8,17,24,25,26], the sensor fusion algorithm requires a nonlinear estimator to estimate both the orientation and the position in the same process, such as Extended Kalman Filter (EKF), Unscented Kalman Filter (UKF), etc. However, in this paper, a multi-rate linear estimator, called “AGOF/Linear Kalman Filter” as our previous work [30], was designed to integrate visual and inertial measurements together without updating orientation information, so that the model can be linear and only needs a small state vector. The following sections briefly review our proposed filter in [30].

### 5.1. State Vector Definition

The state vector $x_{k}$ and the system process noise n can be expressed as follows

(32) $$\begin{array}{ccc} x_{k} & = & {[}_{e}^{c}p_{k};{}_{e}^{c}v_{k};{}_{e}^{c}a_{k};\lambda_{k};b_{a,k}],\\ n & = & [n_{a};n_{\lambda };n_{b_{a}}], \end{array}$$

where ${}_{e}^{c}p_{k}$ is camera position without scale, ${}_{e}^{c}v_{k}$ is camera velocity, ${}_{e}^{c}a_{k}$ is camera acceleration expressed in metric unit (meter), $\lambda_{k}=1/s_{k}$ is the reciprocal of the absolute scale factor, which leads to low-order polynomials and $b_{a,k}$ is the accelerometer bias.

### 5.2. Dynamic Model

The system is assumed to have a uniformly accelerated linear translation at time *k*, so the translation of the camera can be modeled by an equation set. Thus, the dynamic model of the state is defined as follows:

(33) $$\begin{array}{ccc} {}_{e}^{c}p_{k+1} & = & {}_{e}^{c}p_{k}+T\lambda_{k}{}_{e}^{c}v_{k}+\frac{T^{2}\lambda_{k}}{2}{}_{e}^{c}a_{k}+\frac{T^{3}\lambda_{k}}{6}n_{a},\\ {}_{e}^{c}v_{k+1} & = & {}_{e}^{c}v_{k}+T{}_{e}^{c}a_{k}+\frac{T^{2}}{2}n_{a},\\ {}_{e}^{c}a_{k+1} & = & {}_{e}^{c}a_{k}+Tn_{a},\\ \lambda_{k+1} & = & \lambda_{k}+n_{\lambda },\\ b_{a,k+1} & = & b_{a,k}+Tn_{b_{a}}, \end{array}$$

where *T* represents the time span between *k* and k+1. $\lambda_{k}$ is based on a random walk model and the bias $b_{a,k}$ is based on the value and a white noise at time *k*.

### 5.3. Measurement Model

The involved sensors are with two different sampling rates, so two measurements are considered: one is ${}^{s}y_{k}^{m}=\left[{}_{e}^{c}a_{k}^{m}\right]$ when inertial measurements are available and the other is ${}^{c}y_{k}^{m}=\left[{}_{e}^{c}p_{k}^{m}\right]$ when visual measurements are available. Therefore, the updating equation of measurements for output states is:

(34) $$\begin{array}{ccc} y_{k} & = & Hx_{k}+e_{k}, \end{array}$$

with $H_{s,k}=\left(\begin{array}{cccc} 0_{3\times 3} & 0_{3\times 3} & I_{3\times 3} & 0_{3\times 4} \end{array}\right)$ for available inertial measurements or $H_{c,k}=\left(\begin{array}{cccc} \;I_{3\times 3}\; & \;0_{3\times 3}\; & \;0_{3\times 4} & \;0_{3\times 3} \end{array}\right)$ for available visual measurements.

In order to obtain reliable measurements from inertial sensors as the input of measurement model, the effect of the gravity ${}^{e}g={\left[0\;0\;-9.8\right]}^{T}$ denoted in the earth coordinate system *e* should be firstly removed from the raw acceleration measurements ${}^{s}a$ in sensor coordinate system *s* based on the preestimated robust orientation ${}_{e}^{s}{\hat{q}}_{f}$ using the quaternion-based representation. The related equations are depicted in Label (35):

(35) $$\begin{array}{ccc} {}_{e}^{c}a_{k}^{m} & = & R{(}_{s}^{c}q)*\left(R\left({}_{e}^{s}{\hat{q}}_{f,k}\right)*{}^{s}a_{k}-{}^{e}g\right)+{}_{s}^{c}t,\\ {}^{s}a_{k} & = & {}^{s}a_{k}^{m}-b_{a,k}-e_{a,k}, \end{array}$$

where the operator R denotes converting orientation from unit quaternion representation to rotation matrix representation; ${}_{s}^{c}q$ and ${}_{s}^{c}b$ can be obtained from the hand-eye calibration using the method in [34].

## 6. Experimental Results and Analysis

### 6.1. Experimental Setup

**Figure 9:**
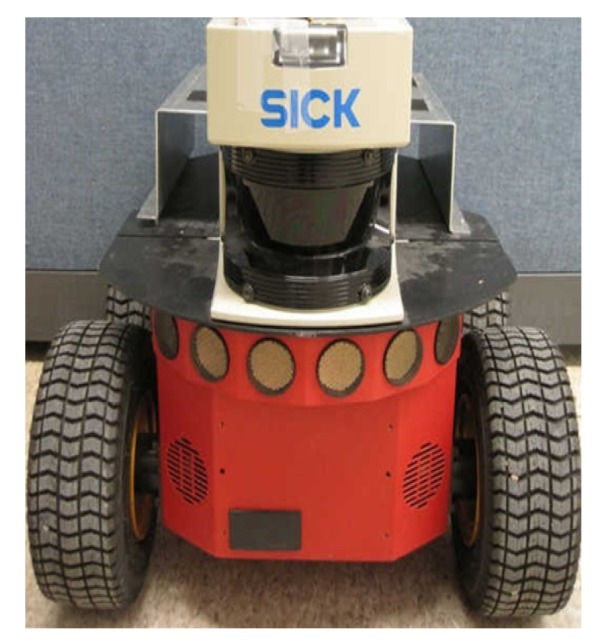
The Pioneer robot platform for experimental illustrations.

The performance of our proposed method was tested by a sunglass with wearable visual-inertial fusion system as shown in Figure 4c in different indoor environments. Firstly, three different experiments were performed in three different indoor environments, which are a hallway, an office room and a home-based environment. In order to test the accuracy of ego-motion estimation, the results from a Pioneer robot were as our ground truth shown in Figure 9. Moreover, the results were compared with those from EKF to verify our proposed MR-LKF more efficient. Secondly, a longer closed-loop path was performed, where a person was walking up and down the stairs with the visual-inertial system. Finally, an office-based dynamic environment was concerned, where a toy car was moving in a straight line.

### 6.2. Experimental Results

#### 6.2.1. Experiment I: Straight-Line Motion Estimation in a Hallway

**Figure 10:**
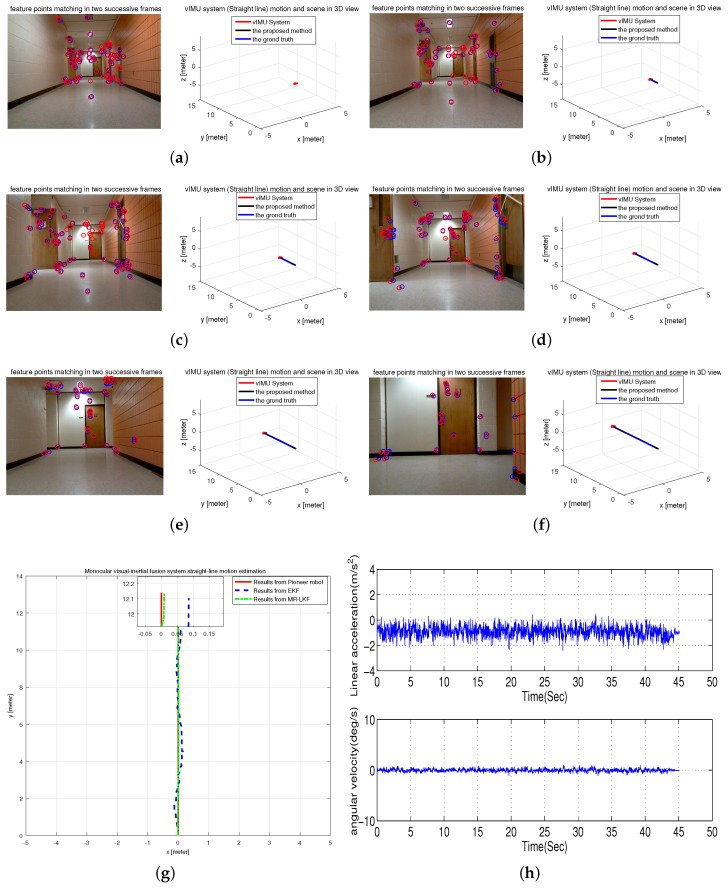
Straight-line Motion Estimation in a hallway. (**a**–**f**) typical frames; (**g**) estimated trajectory with a magnified final position; (**h**) inertial measurements.

The experiment was conducted to attach the proposed vIMU system on the Pioneer robot platform to follow a straight line in our office hallway. The tracked trajectory is shown in Figure 10g compared with the results from EKF and the Pioneer robot. It is clearly seen that the estimated trajectory is more accurate and closer to the ground truth. In addition, Figure 10h shows the inertrial measurements, which obviously shows the movement of the system as slow and smooth. Moreover, typical frames and 3D visualized tracked trajectory are clearly given in Figure 10a–f.

#### 6.2.2. Experiment II: Curve Motion Estimation in an Office Room

**Figure 11:**
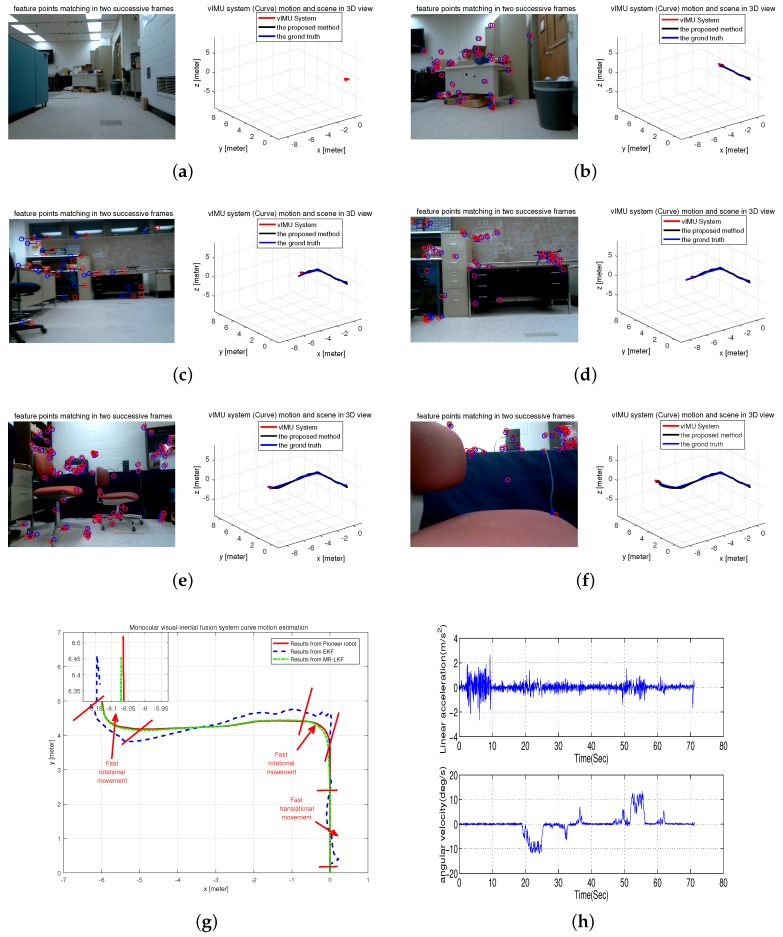
Curve Motion Estimation in an office room. (**a**–**f**) typical frames; (**g**) estimated trajectory with a magnified final position; (**h**) inertial measurements.

In this test scenario, the Pioneer robot attached the visual-inertial system to follow a curve in our office room. Figure 11h shows the inertial measurements, which obviously show that the system experienced fast rotational and translational motion. The tracked trajectory is shown in Figure 11g compared with the results from EKF and the Pioneer robot. It is clearly seen that the estimated trajectory is more accurate and closer to the ground truth. Moreover, typical frames and 3D visualized tracked trajectory are clearly given in Figure 11a–f.

#### 6.2.3. Experiment III: Semicircle Motion Estimation in A Home-Based Environment

**Figure 12:**
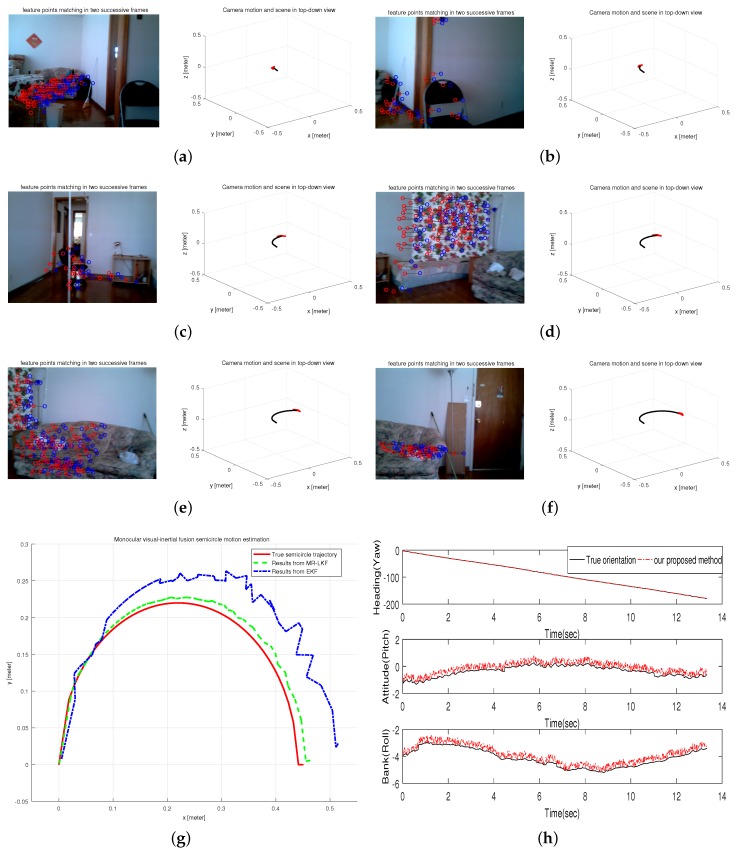
Semicircle Motion Estimation in a home-based environment. (**a**–**f**) typical frames; (**g**) estimated trajectory; (**h**) Orientation estimation.

This test was performed on a controllable robot arm to generate a semicircle movement in a home-based environment. Obviously, the radius of the semicircle is actually the length of the arm, so the accuracy of estimated results can be verified based on the known trajectory equation. The tracked trajectory is shown in Figure 12g compared with the results from EKF and the known trajectory. It is clearly seen that the estimated trajectory is more accurate and closer to the known trajectory. In addition, Figure 12h shows the orientation estimation from our AGOF orientation filter compared with the true orientation. Moreover, typical frames and 3D visualized tracked trajectory are clearly given in Figure 12a–f.

#### 6.2.4. Experiment IV: Closed-Loop Motion Estimation

**Figure 13:**
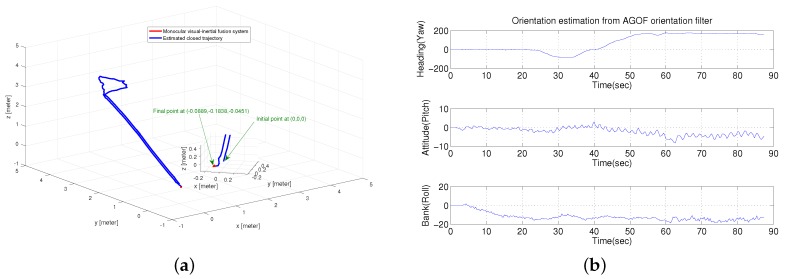
Closed-loop Motion Estimation. (**a**) closed trajectory estimation; (**b**) orientation estimation from AGOF filter.

In this test, a longer trial was performed to verify the efficiency of the proposed method in three-dimensional estimation, where a closed route was conducted by a person walking up and down stairs with the visual-inertial system. Figure 13a shows the estimated trajectory with a magnified final position. It is clearly seen that our proposed method can correct the drift and make the final position very close to the initial position. Moreover, the robust orientation estimation from our AGOF filter, shown in Figure 13b, plays an important role in reducing the drift.

#### 6.2.5. Experiment V: Motion Estimation with Moving Objects in an Office-Based Environment

**Figure 14:**
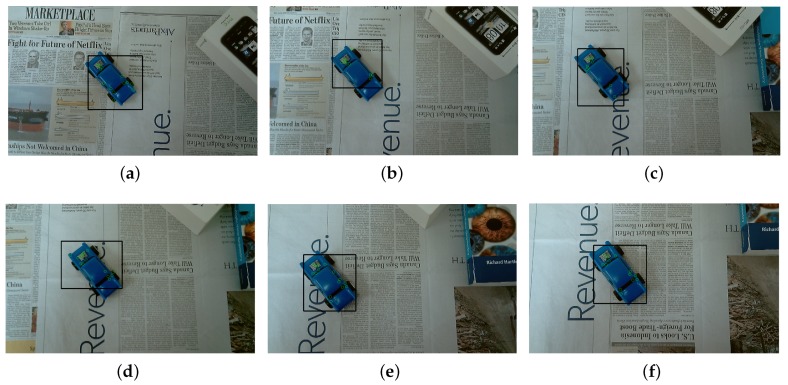

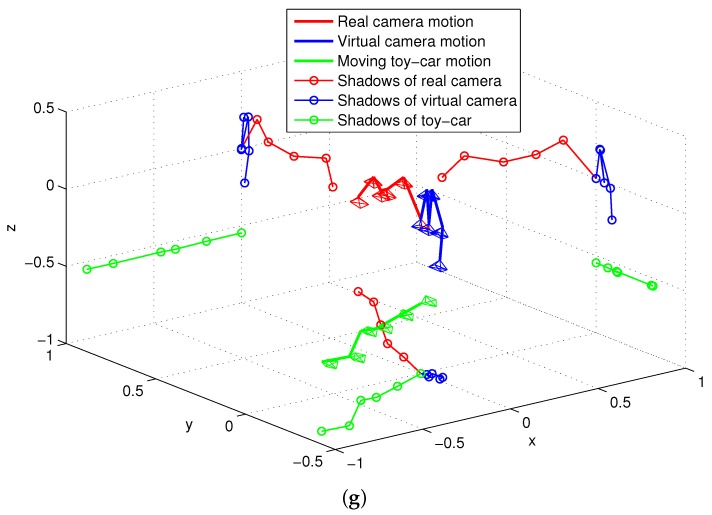
Motion estimation with moving objects in an office-based environment. (**a**–**f**) typical frames; (**g**) the 3D motion of real camera, virtual camera and moving toy car with 2D shadows.

During this test, a person wearing the visual-inertial system was walking in an office-based environment, where a moving toy car was viewed. In this test scenario, a straight line was performed by the moving toy car on a table. The detected moving toy car is labeled within a black bounding box and six key frames with this detected toy car are selected as denoted in Figure 14a–f. Figure 14g shows the motion of the real and virtual camera, which are labeled by using red and blue line, respectively. The motion of moving car is finally derived and labeled by green line in Figure 14g. In particular, the trajectory of a moving car is clearly seen by drawing the shadows of each motion on a 2D plane.

### 6.3. Experimental Analysis

#### 6.3.1. Scale Factor Analysis

**Figure 15:**
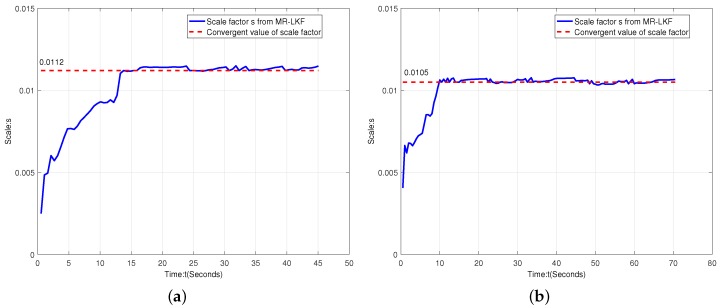
Scale factor analysis. (**a**) scale factor estimation for straight-line motion; (**a**) scale factor estimation for curve motion.

Figure 15 shows the scale factor estimation for straight-line and curve movements. It is clearly illustrated that the scale factor *s* changes over time *t* and its converge time is about 10 s. Therefore, each experiment requires 10 s time calibration at the beginning.

#### 6.3.2. Accuracy Analysis

Four different movements have been used to test the accuracy of our proposed algorithm. For Experiments I and II, the error of each camera position ${}_{e}^{c}p_{k}$ in the reconstructed trajectory is calculated as the Euclidean distance between each point of the estimated camera trajectory and the trajectory $p_{robot,k}$ from Pioneer robot as shown in Label (36). Based on the known trajectory equation of the semicircle, the accuracy can be verified by Label (37), where r=0.221 m is actually the length of robot arm. The accuracy of the fifth experiment is verified based on the known path of the moving toy car:

(36) $$\begin{array}{c} {error}_{k}=\sqrt{{{(}_{e}^{c}p_{k}-p_{robot,k})}^{T}{(}_{e}^{c}p_{k}-p_{robot,k})}, \end{array}$$

(37) $$\begin{array}{ccc} {\left(x-r\right)}^{2}+y^{2} & = & r^{2}. \end{array}$$

Table 3 depicts the error accuracy analysis for four experiments. The true length of four different trajectories is respectively 12 m, 12.5 m, 0.69 m and 1 m. As clearly shown in Figure 10h and Figure 11h, the robot platform experienced different motions with slow and smooth motion in Experiment I and fast rotational and translational motion in Experiment II. From Table 3, it is clearly seen that Experiment I has higher accuracy than Experiment II, but the estimated results from our proposed method in both of Experiments I and II are more accurate than those from the EKF as shown in Figure 10g and Figure 11g.

#### 6.3.3. Dynamic Scene Segmentation Analysis

**Figure 16:**
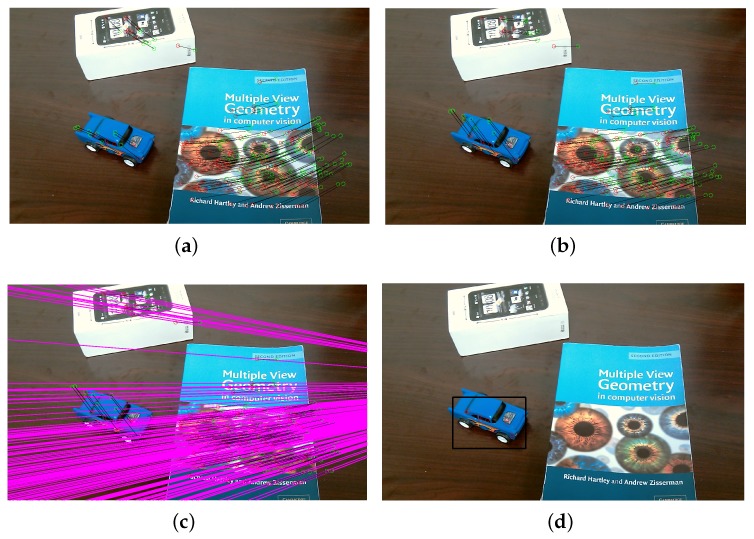
Experimental illustrations for homography recovery. (**a**) detected 2D feature points in first frame; (**b**) detected 2D feature points in second frame; (**c**) sorted feature matches; (**d**) detected toy car.

The experimental illustration was shown in Figure 16 to demonstrate our proposed AGOF-aided homography recovery constraint for dynamic scene segmentation. Figure 16a shows detected 2D feature points and matched in two consecutive frames (green circles in the first frame and red circles in the second frame). In Figure 16b, the feature points in the first frame are transformed and 2D motion paths are obtained based on homography recovery with the help of the AGOF orientation filter. It is clear seen from Figure 16c that the feature matches can be easily sorted out. Finally, the moving object can be detected and separated from the background as denoted in Figure 16d.

**Figure 17:**
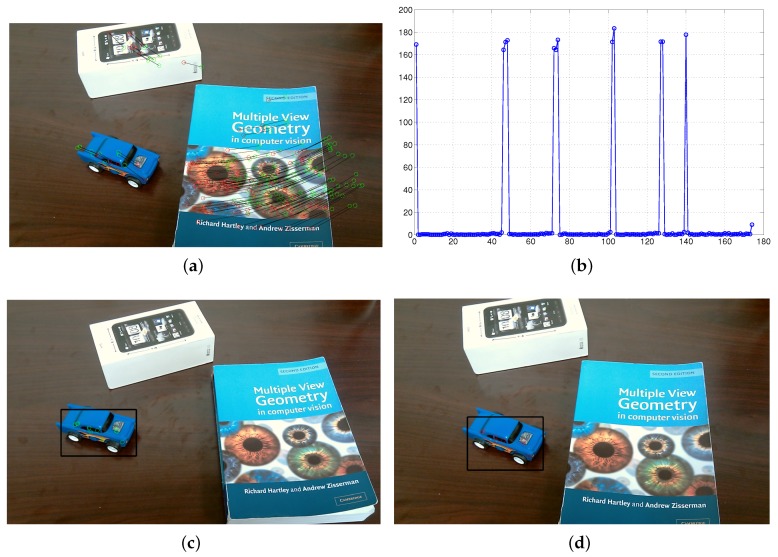
Experimental illustrations for epipolar geometry. (**a**) feature matches; (**b**) distance errors; (**c**) moving toy car detected in first frame; (**d**) moving toy car detected in second frame.

The experimental illustration for the proposed dynamic scene segmentation constrained by epipolar geometry is shown in Figure 17. Figure 17a depicts detected 2D feature points and matched in two consecutive frames. The distance errors between the points and their corresponding epipolar lines are shown in Figure 17b. As we described in Section 4.1.2, the larger the distance is, the more likely the 3D point belongs to an independently moving object. Therefore, the distance errors can be used to sort out the points belonging to the moving object. As a result, the moving object can be separated from the background and tracked in each frame as shown in Figure 17c,d.

#### 6.3.4. Scale Adjustment and Estimation Analysis

**Figure 18:**
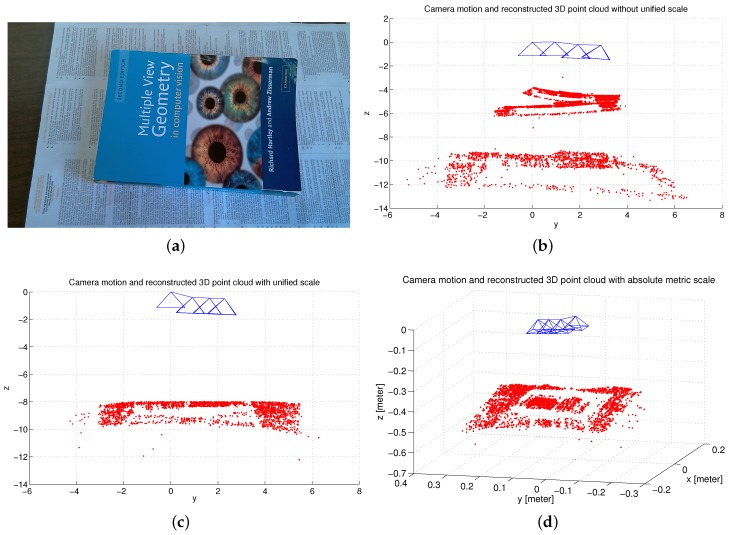
Experimental illustrations for scale adjustment and estimation. (**a**) original frame; (**b**) camera motion and 3D reconstructed point cloud without unified scale; (**c**) camera motion and 3D reconstructed point cloud with unified scale; (**d**) camera motion and 3D reconstructed point cloud with metric scale.

Based on a set of scale inconsistent camera motions and 2D feature matches, the 3D structure could be reconstructed using a linear reconstruction method, such as singular value decomposition (SVD) [10]. While the reconstructed 3D structure could be very noisy and not consistent due to the frame-to-frame reconstruction and the inconsistent estimation of the camera motion. This also results in a duplicated structure as shown in Figure 18b. After adopting our proposed scale adjustment method, a refined 3D point cloud can be obtained with a unified scale. Figure 18c clearly shows that the reconstructed 3D structure is consistent and has no duplicated structure. Having obtained a set of scale consistent camera motions, an absolute global scale can be estimated with the help of the IMU sensor and the 3D reconstructed point cloud with metric scale is shown in Figure 18d.

## 7. Conclusions

A novel wearable absolute ego-motion tracking system was proposed for indoor positioning. The use of pre-estimated orientation from inertial sensors can eliminate mismatched points based on geometry constraints. In addition, a novel concept of “virtual camera” was presented to represent the motion from the motion areas related to each moving object, which was actually the combined motion from the real camera and the moving object. Moreover, an adaptive multi-rare linear Kalman filter was adopted to solve not only scale ambiguity, but also the problem of different sampling rates. This proposed system has much potential to aid the visually impaired and blind people, so, in the future, the goal of our work will aim at several scenarios of real obstacles to test the robustness and effectiveness of the proposed system with motion alerts.

## Figures and Tables

**Table 1:** Related work on inertial-visual fusion (OS, FFT, OFP, OPSP, SE, DFSV, KF, DKF, EKF, UKF, Gyro, Mag and Acc stand for Orientation Source, Fusion Filter Type, Orientation-aided Feature points, Orientation and Position in the Same Process, Scale Estimation, Dimension of Filter’s State Vector, Kalman Filter, Decentralized Kalman Filter, Extended Kalman Filter, Unscented Kalman Filter, Gyroscope, Magnetometer and Accelerometer respectively; x—No and *√*—Yes).

Reference	OS	FFT	OFP	OPSP	SE	DFSV
Chen et al. [24]	vision + Gyro + Acc	EKF	x	x	x	16
. Diel et al. [27]	Gyro	KF	x	x	x	18
Bleser et al. [17]	vision + Gyro + Acc	EKF	x	x	x	16–22
Randeniya et al. [7]	vision + Gyro + Acc	DKF	x	x	x	17
Tardif et al. [8]	vision + Gyro + Acc	EKF	x	x	x	15
Li et al. [25]	vision + Gyro + Acc	EKF	x	x	x	17
Panahandeh et al. [26]	vision + Gyro + Acc	UKF	x	x	x	16
Liu et al. [28]	vision + Gyro + Acc	KF	x	*√*	x	13
This paper	Mag + Gyro + Acc	MR-LKF	*√*	*√*	*√*	13

**Table 2:** Definitions of mathematical symbols and variables.

Symbol	Meaning	Symbol	Meaning
*t*	time	*f*	focal length
*s*	sensor frame	${\left(x,y\right)}^{T}$	2D image point
*c*	camera frame	${\left(X,Y,Z\right)}^{T}$	3D point
*e*	earth frame	${\left(c_{x},c_{y}\right)}^{T}$	camera principal point
${}^{e}g$	gravity in *e*	*K*	camera intrinsic parameter
${}^{s}a$	acceleration in *s*	F	fundamental matrix
${}^{s}\omega$	angular velocity in *s*	E	essential matrix
${}^{s}m$	magnetic field in *s*	b	baseline between two consecutive views
${}_{e}^{s}{\hat{q}}_{f,t}$	final orientation from *s* to *e* at *t*	$R_{1}^{2}$	relative rotation from frame 2 to 1
${}_{s}^{c}q$	relative orientation from *c* to *s*	l	epipolar line
${}_{s}^{c}b$	relative translation from *c* to *s*	e	epipole
${}^{s}\omega_{c,t}$	compensated angular velocity in *s* at *t*	$f_{c}$	sample rate of camera
${}^{s}a_{b,t}$	compensated acceleration in *s* at *t*	λ	reciprocal of the scale factor
$f_{s}$	sample rate of sensor	T	camera ego-motion in homogeneous representation

**Table 3:** Error accuracy analysis in four experiments.

Trajectory Type and Length (m)	Mean Error (m)	Maximum Error (m)	Mean Error over the Trajectory
Experiment I: 12	0.17	0.28	1.42%
Experiment II: 12.5	0.3	0.55	2.4%
Experiment III: 0.69	0.015	0.03	2.2%
Experiment V: 1	0.035	0.12	3.5%

## Supplementary Materials

The following are available online at http://www.mdpi.com/2072-666X/9/3/113/s1, Video S1: Ego-motion demonstration, Video S2: Realtime orientation demonstration.
